# Association of genomic prediction for fertility traits with response to prostaglandin F_2α_ in Holstein cattle

**DOI:** 10.3168/jdsc.2025-0909

**Published:** 2026-03-27

**Authors:** T.G. Guida, S. Borchardt, T.A. Burnett, J.L.M. Vasconcelos, R.L.A. Cerri, A.M.L. Madureira

**Affiliations:** 1 Department of Animal Production, São Paulo State University, Botucatu, Brazil 18168-000; 2 Farm Animal Clinic, Division for Ruminants and Camelids, Unit for Reproduction Medicine and Udder Health, School of Veterinary Medicine, Freie Universitaet Berlin, 14163 Berlin, Germany; 3 University of Guelph, Ridgetown Campus, Ridgetown, ON, Canada N0P 2C0; 4 Applied Animal Biology, Faculty of Land and Food Systems, University of British Columbia, Vancouver, BC, Canada V6T 1Z4; 5 Department of Animal Science, Michigan State University, East Lansing, MI 48824

## Abstract

The objective of this observational study was to evaluate the association between genomic fertility traits, specifically genomic daughter pregnancy rate (gDPR) and genomic cow conception rate (gCCR), and the estrous response following PGF_2α_ administration in lactating dairy cows. Data from 4,147 lactations in 2,743 Holstein cows were collected. At 37 ± 4 DIM, cows with a corpus luteum (≥20 mm; 47.8%; 1,983/4,147) received a single PGF_2α_ injection (25 mg dinoprost tromethamine). Estrous expression was monitored using a collar-mounted automated activity monitor. Cows were classified into gDPR quartiles (first quartile [Q1]: < −1.2; second quartile [Q2]: ≥ −1.2 < −0.3; third quartile [Q3]: ≥ −0.3 < 0.5; and fourth quartile [Q4]: ≥ 0.5). Cows were classified into gCCR quartiles (Q1: < −1.6; Q2: ≥ −1.6 < −0.4; Q3: ≥ −0.4 < 0.8; and Q4: ≥ 0.8). Cows in the lowest gDPR quartile had a longer interval from PGF_2α_ to estrus (88.7 ± 1.9 h) compared with cows in the second, third, and fourth quartiles (83.6 ± 1.8 h vs. 82.1 ± 1.8 h vs. 82.8 ± 1.4 h). A similar pattern was observed for gCCR (Q1 = 89.2 ± 2.0 h vs. Q2 = 81.4 ± 1.9 h vs. Q3 = 83.6 ± 2.0 h vs. Q4 = 83.9 ± 1.9 h). The proportion of cows expressing estrus after PGF_2α_ was lower in the lowest gDPR quartile (49.5% ± 2.2%) than in the highest quartile (59.8% ± 1.9%). Similarly, cows in the lowest gCCR quartile expressed estrus less frequently (52.5% ± 2.3%) compared with the highest quartile (58.0% ± 2.3%). Estrous intensity was greater in cows in the highest quartiles of gDPR and gCCR, whereas estrus duration (13.7 ± 3.7 h) was not associated with genomic fertility traits. Parity and BCS also influenced estrous outcomes, with first-lactation cows and those with moderate BCS (≥3.5) exhibiting greater estrous response. In conclusion, genomic fertility traits were associated with differences in estrous dynamics following PGF_2α_ administration in lactating dairy cows. This study provides needed evidence in lactating cows, rather than heifers, demonstrating that genomic fertility traits are directly linked to postpartum estrous physiology and behavioral expression. Integration of genomic fertility traits into reproductive management decisions may improve estrus detection and overall reproductive efficiency in dairy herds.

## Highlights

• Genomic fertility merit was not associated with the proportion of CL at 37 ± 4 days in milk (DIM).
• Cows with greater genomic merit were associated with stronger estrous expression.
• Cows with low fertility traits showed delayed or no estrus after PGF administration.

## Graphical Abstract

**Figure:**
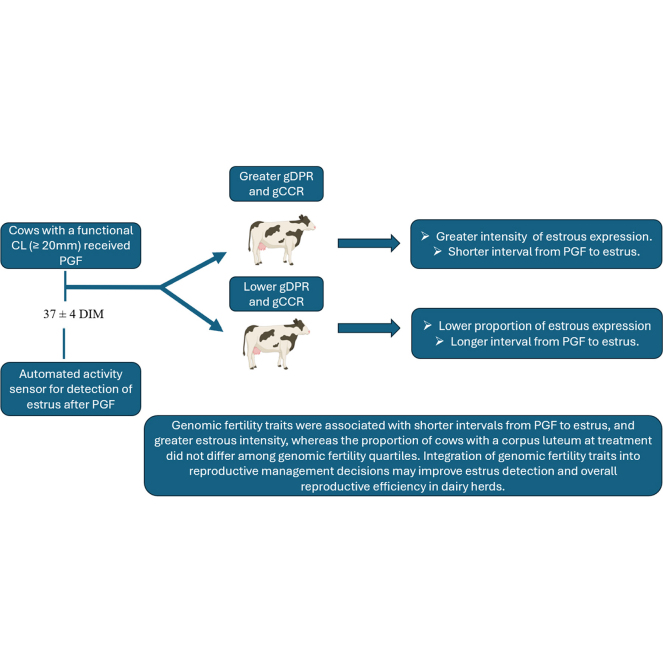
**Summary:**The objective of this study was to evaluate the association between genomic predictions for fertility traits and the response to prostaglandin F_2α_ (PGF_2α_) in lactating Holstein cows. Using data from 4,147 lactations (n = 2,743 lactating cows), we evaluated how genomic daughter pregnancy rate (gDPR) and genomic cow conception rate (gCCR) influenced estrous expression after PGF administration. The proportion of cows with a corpus luteum (CL) at treatment did not differ among genomic fertility quartiles. However, cows with higher genomic fertility merit exhibited shorter intervals from PGF to estrus and showed greater intensity of estrous behavior compared with cows of lower fertility merit. In contrast, cows in the lowest quartiles of gDPR and gCCR were less likely to respond to PGF and displayed weaker estrous expression. These results demonstrate that genomic fertility traits are associated with variation in estrous response following PGF administration, suggesting that genomic selection can guide more effective, tailored reproductive strategies in dairy herds, improving both fertility outcomes and overall herd productivity.

Reproduction is a fundamental aspect of dairy farming because it directly affects productivity, reproductive efficiency, and overall profitability. There has been a major concern regarding the decline in reproductive performance in Holstein cows from the late 1950s until the year 2000 (Lucy, 2001). Factors contributing to this decline can be multifaceted and may include genetic, environmental, nutritional, and management-related issues (Lucy, 2001). It is believed that the intense selection for milk yield with disregard to functional traits such as fertility have negatively affected the reproductive performance of Holstein cattle. As a result, a fertility trait, genomic daughter pregnancy rate (**gDPR**) was introduced in early 2000s (VanRaden et al., 2004). Since then, the dairy industry has shifted toward incorporating fertility and health traits into genetic selection, alongside the adoption of precision technologies and enhanced management practices, resulting in gradual improvements in reproductive performance (Fricke and Wiltbank, 2022; Brito et al., 2025). Genomic daughter pregnancy rate is a genomic estimate of reproductive efficiency in dairy cows. Although gDPR predicts the likelihood that a bull's daughter will become pregnant after calving, the genomic cow conception rate (**gCCR**) estimates the probability that a bull's daughters will conceive following artificial insemination (**AI**). However, studies investigating fertility outcomes specifically in relation to gCCR remain limited. The recent advancements in genomic prediction for gDPR have opened new possibilities for using it as a selection criterion to improve the fertility of lactating dairy cows. Several recent studies, including those by Veronese et al. (2019a), Lima et al. (2020), and Madureira et al. (2022b), have demonstrated that gDPR can be effectively employed as a tool to enhance fertility. Norman et al. (2009) further observed that integrating gDPR into reproductive programs successfully reversed the phenotypic decline in dairy cow fertility. A recent study by Melo et al. (2025), demonstrated that cows with greater gCCR had better reproductive performance, including shorter intervals from calving to first service, fewer services to conception, and higher rates of AI at estrus and pregnancy at first service, compared with cows with lower gCCR. Unlike gDPR, there were no interactions between reproductive programs and gCCR, indicating that cows with greater gCCR consistently performed better regardless of the reproductive strategy used. These findings highlight the potential of incorporating genomic data into reproductive programs to achieve better reproductive performance in dairy herds. The occurrence of estrous expression holds significant importance in dairy cattle management because it leads to increased reproductive efficiency, reduced days open, enhanced pregnancy rates, and decreased reproductive costs. Occurrence and intensity of estrus have positive effects on fertility in both spontaneous estrus (Burnett et al., 2018; Tippenhauer et al., 2021a,b) and timed AI programs (Pereira et al., 2016; Madureira et al., 2019). Moreover, in recipient cows, the occurrence of estrus has been linked to increased pregnancy rates per embryo transfer, whether using estradiol (**E2**)-based (Pereira et al., 2016; Madureira et al., 2022a) or GnRH-based protocols (Jinks et al., 2013). Additionally, estrus occurrence and intensity has been associated with reduced pregnancy loss (Pereira et al., 2016; Madureira et al., 2022a).

Genomic daughter pregnancy rate was positively associated with the duration of and the likelihood of activity peak (0 = no estrus, 100 = maximum activity) ≥80 and was negatively associated with rumination nadir at PGF_2α_-induced estrus using dairy heifers (Veronese et al., 2019a). In a companion study, these authors reported that heifers with greater gDPR had larger ovulatory follicles and greater concentrations of E2 at estrus after PGF_2α_ administration (Veronese et al., 2019b). The authors argued that cattle with elevated genetic merit for fertility traits are more likely to have estrus behavior and preovulatory hormonal profile that are commonly associated with greater likelihood of pregnancy establishment and maintenance. Although it has been shown that estrous expression is an important trait for fertility in dairy heifers, limited evidence exists for an association of gDPR and gCCR on estrous expression after PGF_2α_ administration in lactating dairy cows. Thus, the objective of this study was to evaluate the association between gDPR and gCCR with the response of PGF_2α_, specifically focusing on the occurrence and intensity of subsequent estrous expression among lactating dairy cows that had corpus luteum (**CL**) at 37 ± 4 DIM and received PGF_2α_. We hypothesized that cows with greater genomic fertility traits (i.e., gDPR and gCCR) would exhibit more intense estrous responses following PGF_2α_ administration.

This observational study was conducted in a commercial farm in the state of Minas Gerais, Brazil (latitude 19°31′08″; longitude 46°04′90″) from January 2019 to December 2021. All animals that were previously genotyped on the farm (from 2015 to 2017) were enrolled in this study. The local Institutional Animal Care Committee (Animal Ethics and Experimentation Committee of the Faculty of Veterinary Medicine and Animal Science [FMVZ], UNESP-Botucatu), following the requirements of the practices outlined in the Guide for the Care and Use of Agricultural Animals in Research and Teaching, published by the Federation of Animal Science Societies (FASS, 2010), approved all experimental procedures. Lactating cows were housed in a naturally ventilated freestall barn that contained stalls lined with rubber bedding and a layer of sawdust. Cows were fed thrice daily a TMR that was formulated to meet or exceed the requirements of a lactating Holstein cow (NRC, 2001). Water and TMR were available ad libitum.

Hair samples were collected from the tail switch of each animal, primarily when they were around 2 mo old. These samples were stored in a provided sample collector until further analysis. Subsequently, the samples were genotyped using commercially available SNP genotyping platforms (Clarifide, Zoetis, São Paulo, SP, Brazil). The interpretation of gDPR and gCCR results were based on a ranking database derived from genotyping. Genomic daughter pregnancy rate serves as an indicator of the animal's genetic potential for improved reproductive efficiency and is calculated by considering the days open of a bull's daughters. Cows had their ovaries examined by transrectal ultrasonography (Aloka SSD-500, Aloka Co. Ltd., Wallingford, CT) using a 7.5-MHz linear rectal transducer at 37 ± 4 DIM and were enrolled in a presynchronization program. At this time, all cows received a single intramuscular injection of dinoprost tromethamine (PGF_2α_; 25 mg; 5.0 mL of Lutalyse, Zoetis, São Paulo, Brazil). Because PGF_2α_ was administered only to cows with a CL ≥ 20 mm, subsequent analyses of estrous response were restricted to cyclic cows with luteal activity at 37 ± 4 DIM. This PGF_2α_ treatment was part of a modified presynchronization strategy routinely implemented on the farm to promote early luteal turnover; however, the present study specifically evaluated the estrous response to this single PGF_2α_ injection, and subsequent synchronization steps were not included in the analysis during a 7-d post-treatment observation window.

All cows were equipped with a collar-mounted automated activity monitor (**AAM**; Smarttag Neck, Nedap Livestock Management, Groenlo, the Netherlands), which has been validated for estrus detection in lactating dairy cows Roelofs et al. (2017). This accelerometer recorded activity data in real time. The relative change in activity was measured using a z-score transformation and a proprietary algorithm. The z-score represents the deviation of the current activity from the mean activity within a certain period (i.e., comparing the number of neck movements within every 2-h period with the same 2-h period of the preceding 10 d for each cow). The baseline used for this comparison is updated on a rolling basis, allowing the system to continuously adjust for each cow's changing activity patterns over time. When the z-score exceeds a threshold for multiple consecutive periods, a cow is considered in estrus and an AAM alert (i.e., attention) is generated. Values for attention could be 0 (no estrus) or 1 (estrus). Onset of estrus was defined to occur at the first attention changed from 0 to 1. The end of estrus was defined by the first instance at which the attention changed from 1 to 0. In summary, an estrus event was defined as attention change from 0 to 1 for more than 2 time periods (5 h) based on a proprietary algorithm. The choice of 5 h accounts for algorithm-specific smoothing and filtering of short, transient activity spikes, ensuring that brief, nonestrus-related movements do not trigger a false alert. For each estrus event, peak activity and estrus duration were determined. Estrus intensity was quantified using the “x-factor,” a proprietary metric derived from the z-score that scales relative activity within the estrus event. The x-factor is unitless, with typical thresholds validated in lactating cows (Roelofs et al., 2017), and corresponds to the magnitude of deviation from baseline activity. Maximum x-factor values within each estrus event were used to represent estrus intensity. Estrous duration was defined as the interval from onset to end of an estrus event. The interval from the administration of PGF_2α_ to estrus was calculated based on the time of PGF_2α_ injection, which was administered at 37 ± 4 DIM, approximately at 9:00 a.m. This interval was measured from the time of PGF_2α_ administration until the onset of increased activity, indicating the beginning of an estrus event.

Means, SD, distributions, and normality tests were obtained using the Univariate procedure of SAS University Edition (SAS Institute Inc., Cary, NC). Parity was classified into first lactation, second lactation, and third or greater lactations. Body condition score was classified as low (≤2.75), medium (≥3.0 to ≤3.25), and moderate (≥3.5), based on the 33rd and 66th percentiles, with cows assigned to mutually exclusive tertiles. Cows were classified into gDPR quartiles (first quartile [**Q1**]: < −1.2; second quartile [**Q2**]: ≥ −1.2 < −0.3; third quartile [**Q3**]: ≥ −0.3 < 0.5; and fourth quartile [**Q4**]: ≥ 0.5). Cows were classified into gCCR quartiles (Q1: < −1.6; Q2: ≥ −1.6 < −0.4; Q3: ≥ −0.4 < 0.8 and Q4: ≥ 0.8). Quartiles for gDPR and gCCR were defined based on the distribution of genotyped cows within the study herd, and therefore represent relative fertility rankings specific to this population rather than absolute thresholds from the national genomic base. The interval from PGF_2α_ to estrus, as well the intensity and duration of estrus, were analyzed as continuous dependent variables using ANOVA with the linear mixed model procedure. Separate models were constructed to evaluate associations with either gDPR or gCCR, along with parity, BCS, DIM, and milk production (milk yield at 60 DIM). Presence of a CL at 37 ± 4 DIM was modeled across all lactations using mixed-effects logistic regression with gDPR or gCCR quartile as a fixed effect and cow as a random effect to account for repeated lactations; results are presented as odds ratios (**OR**) with 95% CI. Expression of estrus (estrus or no estrus) was used as binomial dependent variable and tested for the association with either gDPR or gCCR (different models, due to its strong correlation), parity, BCS, DIM, and milk production, using a mixed-effects logistic regression procedure by specifying the distribution as binary. To examine the relationship between milk production and the likelihood of estrus expression following PGF_2α_ treatment, we fitted a mixed-effects logistic regression model that included both linear and quadratic terms for milk production. Parity (first, second, and third or greater lactation) was included as a stratification factor, and cow was included as a random effect to account for repeated measurements. In all models, cow was considered the experimental unit and as a random effect, lactation as the repeated measure using first-order antedependence as the covariance structure. Lactation was used as a repeated measure to account for the fact that observations from cows enrolled in different lactations were not independent from each other. For all models, only variables with a *P*-value <0.15 were kept in final models, using manual backward elimination. Differences between groups with *P* ≤ 0.05 were considered significant and those between 0.05 > *P* ≤ 0.10 were designated as a tendency, when using Tukey's adjustments. All multivariable models were constructed using the variables as described, and manual backward stepwise elimination was used. Interactions were tested between all variables selected in the final model and kept if *P* < 0.05.

Data from 4,147 lactations from 2,743 lactating dairy cows were enrolled in the study. The gDPR and gCCR were available for all lactating dairy cows. Cows that were considered as suspected cystic (i.e., follicular structure ≤25 mm), had no CL, or were missing data at 37 ± 4 DIM were excluded from analyses (6.0% [248/ 4,147]; 39.7% [1,646/4,147]; and 1.1% [45/4,147], respectively). Only cows that had a CL (≥20 mm) at 37 ± 4 DIM were kept in the study (47.8% [1,983/4,147]). When CL presence at 37 ± 4 DIM was evaluated across the full population of lactations (n = 4,147), there was no significant difference (*P* = 0.70) in the likelihood of having a CL among gDPR quartiles (Q1 = 45.7% [428/937], Q2 = 48.0% [477/993], Q3 = 47.9% [428/893], Q4 = 49.1% [650/1,324]). The odds of having a CL tended to be greater in Q4 compared with Q1 (OR = 1.14; 95% CI: 0.97–1.33; *P* = 0.10). Similarly, there was no significant difference (*P* = 0.70) in the likelihood of having a CL among gCCR quartiles (Q1 = 45.3% [425/937], Q2 = 47.3% [470/993], Q3 = 48.3% [431/893], and Q4 = 49.6% [657/1,324]). The likelihood of CL presence tended to be greater in Q4 than Q1 (OR = 1.16; 95% CI: 0.99–1.36; *P* = 0.07). This analysis therefore describes the association between genomic fertility quartiles and the probability of being eligible for PGF_2α_ treatment based on the presence of a CL at 37 ± 4 DIM. Mean (±SD) milk production was 47.8 (±9.1) kg/d with the minimum of 11.3 kg/d and the maximum of 77.3 kg/day. Mean ± (SD) genomic daughter pregnancy rate was −0.20 ± (1.4) with the minimum of −5.2 and the maximum of 5.5. Mean ± (SD) genomic cow conception rate was −0.38 (1.7) with the minimum of −6.3 and the maximum of 5.3. There was a strong correlation between gDPR and gCCR (R^2^ = 0.76; Pearson r = 0.88; < 0.0001)

Cows in the first-lactation group had shorter interval from PGF_2α_ to estrus (79.2 ± 1.5 h, *P* < 0.01) compared with cows in the second (84.5 ± 1.5 h) and third or greater lactation groups (91.5 ± 1.6 h). There was a difference between second and third lactation (*P* = 0.03). Cows with lower BCS had longer interval from PGF_2α_ to estrus compared with cows with moderate BCS (88.3 ± 2.0 h vs. 82.5 ± 1.2 h, respectively, *P* = 0.05). There was no difference in the interval from PGF_2α_ to estrus between cows with medium and moderate BCS (*P* = 0.48). Cows with lower gDPR (Q1) had longer interval from PGF_2α_ to estrus compared with cows on Q2, Q3, and Q4, as shown in Table 1. No significant differences were observed among Q2, Q3, and Q4. Similarly, cows in the lower quartile for gCCR experienced a longer interval from PGF_2α_ to estrus compared with cows in the other quartiles (Q2, Q3, and Q4, Table 1). There was no interaction between gDPR and parity regarding the interval between the administration of PGF_2α_ and estrus detected by the AAM (*P* = 0.92). Similarly, for gCCR, no significant interaction with parity was observed (*P* = 0.78). Milk yield at d 60 was associated with the interval from PGF_2α_ to estrus (*P* = 0.02). There was a quadratic relationship between milk production and the proportion of cows expressing estrus post-PGF_2α_ injection, with both low- and high-producing cows showing a lower proportion of estrous expression (*P* = 0.04; Figure 1). Additionally, no interaction was observed between milk production, estrous expression, and parity (*P* = 0.83). Cows classified with lower intensity of estrus had longer interval from PGF_2α_ to estrus compared with cows classified as having greater intensity of estrus (95.5 ± 4.9 h vs. 84.3 ± 1.2 h; *P* = 0.02). Shorter estrus duration was associated with longer interval from PGF_2α_ to estrus compared with cows with longer estrus duration (86.9 ± 1.4 h vs. 83.0 ± 1.1; *P* = 0.03).

**Table 1:** Interval from PGF_2α_ to estrus and the proportion of cows showing estrus after PGF_2α_ according to the gDPR and gCCR

Fertility trait	n^1^	Interval from PGF_2α_ to estrus^2^ (h)	Proportion of estrus after PGF_2α_ (%)
gDPR			
Q1	428	88.7 ± 1.9^a^	49.5 ± 2.2^a^
Q2	477	83.6 ± 1.8^ab^	55.4 ± 2.4^ab^
Q3	428	82.1 ± 1.8^b^	54.3 ± 2.4^ab^
Q4	650	82.8 ± 1.4^b^	59.8 ± 1.9^b^
gCCR			
Q1	425	89.2 ± 2.0^a^	52.5 ± 2.3^a^
Q2	470	81.4 ± 1.9^b^	52.8 ± 2.3^a^
Q3	431	83.6 ± 2.0^b^	55.8 ± 2.4^ab^
Q4	657	83.9 ± 1.9^b^	58.0 ± 2.3^b^

a,b Different letters indicate differences between variables within the columns (*P* < 0.05).

1 n represents the number of lactations included in the estrous response analyses and corresponds only to cows with a confirmed CL (≥20 mm) at 37 ± 4 DIM that received PGF_2α_.

2 The interval from PGF_2α_ to estrus was measured from the time of PGF_2α_ administration until the onset of increased activity, indicating the beginning of an estrus event. Estimated marginal means (±SE) from linear mixed-effects models.

**Figure 1:**
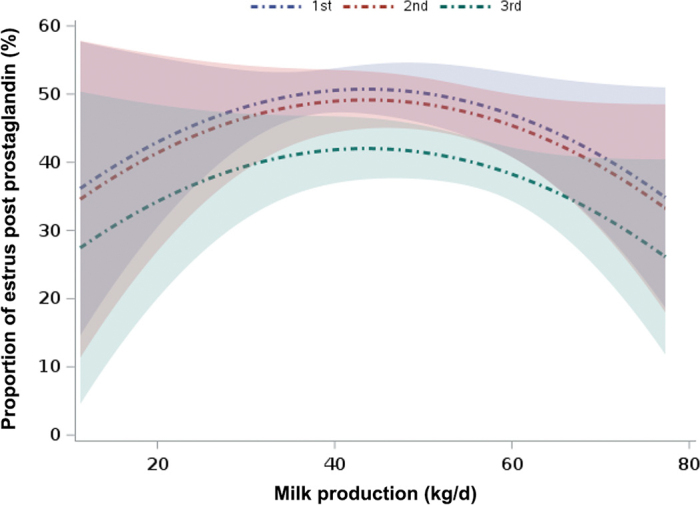
Predicted proportion of cows expressing estrus after PGF_2α_ treatment in relation to milk production (kg/d) at d 60, detected by an AAM. Estimates were derived from a mixed-effects logistic regression model stratified by parity: first lactation (1st), second lactation (2nd), and third or greater lactation (3rd). Shaded areas represent the 95% CI around the predicted probabilities for each parity group.

Cows in the first-lactation group had greater proportion of estrus after PGF_2α_ compared with cows in the second and third or greater lactation group (46.6% ± 1.4% vs. 42.0% ± 1.5% vs. 34.4% ± 1.4%; *P* < 0.001 respectively). There was also a significant difference (*P* = 0.01) between cows in the second and third or greater lactations. Cows classified as moderate BCS had greater proportion of estrus after PGF_2α_ compared with cows classified as thin and medium BCS (45.9% ± 1.3% vs. 39.9% ± 1.4% vs. 32.9% ± 1.8%; *P* < 0.001, respectively). Cows classified as thin had a greater likelihood of estrus compared with cows having medium BCS (*P* = 0.02). There was an association of gDPR and cows expressing estrus after PGF_2α_ (*P* = 0.03). A lower proportion of cows in the lowest gDPR quartile (Q1) expressed estrus after PGF_2α_ compared with cows in the greater gDPR quartile (Q4), as shown in Table 1. Similarly, gCCR showed comparable results, with a lower proportion of cows in the lowest quartiles (Q1 and Q2) expressing estrus (*P* = 0.04, Table 1). Mean (±SD) for the intensity and duration of estrus was 12.6  ±  12.7 x-factor and 13.7  ±  3.7 h, respectively. Median (interquartile range) values were 10.5 (6.2–17.8) for estrus intensity and 13.0 (11.0–16.5) h for estrus duration. First and second lactation cows had greater intensity of estrus compared with cows on the third or greater lactations (15.3 ± 0.5 vs. 16.4 ± 0.6 vs. 14.1 ± 0.6 x-factor, *P* = 0.01, respectively). Duration was not associated with parity (*P* = 0.27). Thin cows had lower intensity of estrus after PGF_2α_ compared with cows with medium and moderate BCS (13.5 ± 0.7 vs. 15.1 ± 0.5 vs. 16.2 ± 0.4 x-factor; *P* < 0.01, respectively). There was no association between duration of estrus after PGF_2α_ and BCS (*P* = 0.41). There was no association between milk production and the duration (*P* = 0.78) or intensity of estrus (*P* = 0.31). Cows classified in Q4 of gDPR exhibited a greater intensity of estrus after PGF_2α_ compared with cows in the other quartiles (Q1: 14.1 ± 0.6, Q2: 14.8 ± 0.6, Q3: 15.5 ± 0.7, Q4: 16.4 ± 0.5 x-factor, *P* = 0.04). Similarly, cows in the highest quartile of gCCR demonstrated a greater intensity of estrus after PGF_2α_ compared with cows in the other quartiles (Q1: 14.7 ± 0.7, Q2: 14.9 ± 0.7, Q3: 14.5 ± 0.7, Q4: 16.8 ± 0.5 x-factor, *P* = 0.02). No significant association was found between the duration of estrus after PGF_2α_ and gDPR (*P* = 0.12) or gCCR (*P* = 0.61). The objective of this observational study was to evaluate the association between genomic fertility traits, gDPR and gCCR, with the response to PGF_2α_, specifically focusing on the occurrence and intensity of subsequent estrous expression in lactating dairy cows. Our findings demonstrated that among cows with a CL at 37 ± 4 DIM that received PGF_2α_, those with higher gDPR and gCCR exhibited shorter intervals to estrus and greater estrous intensity compared with cows with lower genomic fertility merit.

In contrast, cows with lower gDPR and gCCR, had a delayed response to PGF_2α_, and showed reduced intensity or absence of estrous behavior. These results suggest that genomic fertility traits are strongly associated with reproductive responses following PGF_2α_, which might be linked with different biological processes such as luteal regression and follicular development, which directly affects estrous expression and ovulation response. The cows used in this research were genotyped in 2015 through 2017 and later followed between 2019 and 2021 within a single commercial herd in Brazil, which may limit external validity and generalization to broader production systems. Genotyped animals may differ systematically from nongenotyped herd mates due to historical selection for fertility, production, or management value, and thus may not reflect the genetic distribution of an average commercial population. Furthermore, because outcome analyses required cows to have a CL at 37 ± 4 DIM, the exclusion of cows without luteal activity likely removed a disproportionate number of low-fertility animals, narrowing the analytical population and potentially underestimating the true magnitude of difference between genomic fertility quartiles. Conditioning on CL presence also introduces a form of selection bias when interpreting estrous response after PGF_2α_ because cows failing to form a CL, arguably the most reproductively vulnerable group, were not retained for comparison. These limitations should be considered when extrapolating results, and future studies including both cyclic and anovular cows across diverse herds will be valuable for validating and expanding the applicability of these findings. Therefore, the associations between genomic fertility traits and estrous response reported here should be interpreted as conditional on cows being cyclic at the time of PGF_2α_ administration.

The physiological basis for these associations likely lies in the function of the hypothalamic–pituitary–gonadal axis and the dynamics of luteal tissue. Cows with poor fertility merit tend to exhibit altered endocrine profiles, including lower E2 concentrations at estrus and prolonged luteal phases (Cummins et al., 2012a), which may hinder luteolysis and estrous behavior. In our study, cows in the lowest gDPR or gCCR quartiles exhibited delayed or weaker estrous responses, suggesting less effective luteal regression or suboptimal follicular development, although these physiological processes were not directly assessed. In contrast, genetically superior cows showed earlier and more intense responses to PGF_2α_, consistent with more coordinated luteal and follicular dynamics, again acknowledging that these mechanisms were not directly measured.

These findings align with previous research in both heifers and cows. Veronese et al. (2019a) reported that heifers with higher gDPR exhibited shorter intervals from PGF_2α_ to estrus and more intense estrous activity. In a companion study, Veronese et al. (2019b) found that these animals also had larger ovulatory follicles and higher circulating E2, suggesting improved follicular development and hypothalamic responsiveness. Similarly, Cummins et al. (2012b) and Moore et al. (2014) observed that cows with higher genetic fertility merit showed increased estrous activity and stronger luteal profiles, which can enhance the success of estrus-based reproductive protocols. In our study, estrus was detected using an AAM system, and all cows received a single PGF_2α_ dose. Despite this simplified protocol, approximately 41.9% of cows exhibited estrus, which is lower than rates reported in more intensive protocols such as Presynch-Ovsynch. Nevertheless, cows with superior gDPR and gCCR were more likely to express estrus and with greater intensity, underscoring the role of genetic fertility merit in enhancing responsiveness even under minimal hormonal intervention. Genetic and environmental interactions may also influence estrous response. Sitko et al. (2023) reported a significant interaction between season and gDPR in cows undergoing AI after a single PGF_2α_ dose, highlighting the importance of both genomic and environmental factors. This suggests that cows with higher fertility merit may be better suited for simplified, estrus-based reproductive programs, whereas cows with lower genetic merit may require more controlled timed AI protocols to compensate for physiological limitations; however, data from Sitko et al. (2023) indicate that double-Ovsynch protocols may provide superior results irrespective of fertility group. From a physiological perspective, differential expression of genes involved in PGF_2α_ synthesis and luteal sensitivity may help explain some of the observed differences in estrous response, although causality cannot be confirmed in this study. Moore et al. (2016) reported that cows with low fertility exhibited increased expression of genes involved in PGF_2α_ synthesis and CL sensitivity to PGF_2α_, potentially altering the timing of luteolysis and ovulation. Although a stronger PGF_2α_ response could theoretically lead to shorter intervals to estrus, the overall hormonal environment, including E2 synthesis, follicular dynamics, and feedback sensitivity, may influence the timing and intensity of estrous behavior. Supporting this, Cummins et al. (2012b) found that cows with lower fertility had a shorter interval from peak activity to ovulation, suggesting an asynchrony of behavior and ovulatory timing that could impair detection and AI success. Importantly, gDPR and gCCR are highly correlated (r = 0.88), yet in our analysis, neither trait interacted significantly with parity or milk production, suggesting that these genomic predictors are robust across various physiological states and management conditions. This adds further support to their use as practical tools in reproductive management. In conclusion, this study reinforces the value of integrating genomic, physiological, and behavioral data to improve reproductive management in lactating dairy cows. Specifically, genomic fertility traits, particularly gDPR and gCCR, proved to be reliable predictors of patterns of estrous behavior following PGF_2α_ administration. Cows with higher genomic fertility merit exhibited stronger responses to estrus-based synchronization protocols, characterized by shorter intervals from PGF_2α_ to estrus, greater estrus intensity, and a greater likelihood of estrous expression. These findings suggest that genomic selection may help inform more tailored, cow-specific reproductive strategies. Genetically superior cows may respond well to estrus-based protocols, while those with lower fertility merit may require synchronization protocol approaches to overcome underlying physiological limitations. By considering genomic fertility traits alongside other key factors such as parity, BCS, and milk production, producers can make more informed, data-driven decisions that enhance reproductive efficiency and sustainability in modern dairy systems. Future research should continue to explore how genomic information can be leveraged to refine estrus detection technologies and reproductive protocols across diverse environmental and management conditions.
